# A step closer to fully understand how the engine of life is repaired from damages caused by its fuel

**DOI:** 10.1093/plphys/kiad417

**Published:** 2023-08-03

**Authors:** Henning Kirst

**Affiliations:** Assistant Features Editor, Plant Physiology, American Society of Plant Biologists, USA; Departamento de Genética, Campus de Excelencia Internacional Agroalimentario ceiA3, Universidad de Córdoba, Córdoba, 14071, Spain; Instituto Maimónides de Investigación Biomédica de Córdoba (IMIBIC), Córdoba, 14004, Spain

Oxygenic photosynthesis utilizes the energy of sunlight and converts it into chemical energy through charge separation at the photochemical reaction centers (Fischer et al. 2016; Nelson and Ben-Shem 2004). This energy is processed through the electron transport in the photosynthetic active thylakoid membrane and is eventually delivered in the form of reductant (reduced ferredoxin) and high-energy phosphate bonds (ATP), while water is oxidized, releasing oxygen. However, the released oxygen also has an intrinsic toxic effect on the photosynthetic organism because the light energy that is absorbed by the pigments can be transferred onto oxygen, which leads to the formation of reactive oxygen species (ROS) (Krieger-Liszkay 2004; Rutherford et al. 2012). These are extremely reactive compounds that damage the pigments themselves and other biological materials such as proteins that can ultimately lead to cell death. Thus, photosynthetic organisms have evolved a whole array of mechanisms that protect the cell from the generation of ROS summarized as nonphotochemical quenching processes (Müller et al. 2001).

**Figure 1.**
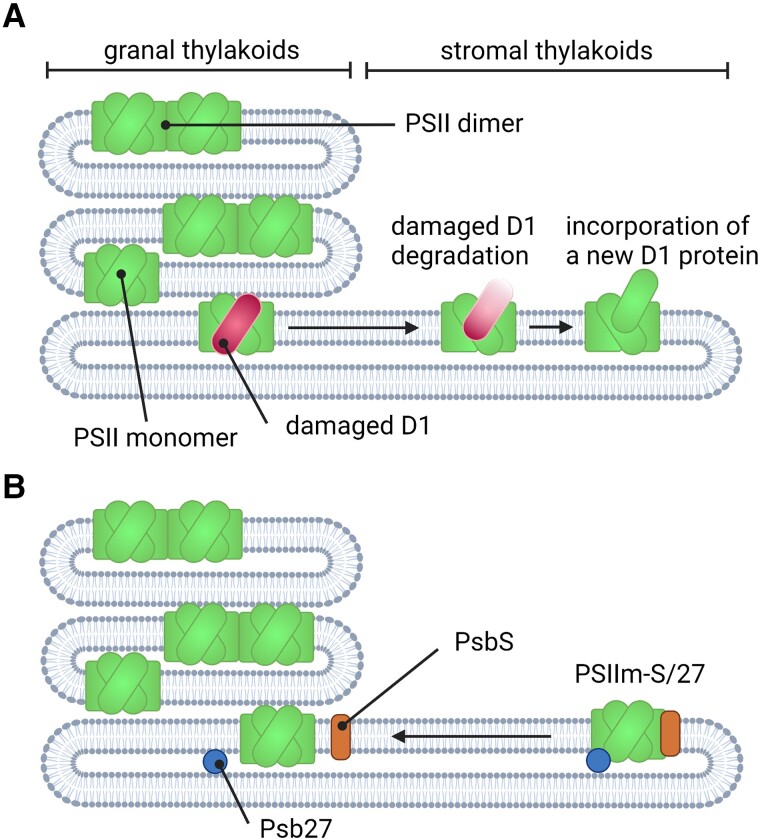
Simplified schematic of the PSII repair cycle. **A)** PSII with a damaged D1 protein gets transferred from the granal to the stromal region of the thylakoid membrane, where the damaged D1 is degraded and a new, freshly translated D1 is incorporated into the PSII complex. **B)** PsbS and Psb27 bound to the repaired PSII monomer (PSIIm-S/27) protect the cell from photodamage by switching off the PSII while in transit back to the granal region, where PsbS and Psb27 detach from the complex triggered by bicarbonate to activate the PSII.

Nevertheless, ROS formation is particularly problematic for the Photosystem II because it is the machinery that releases molecular oxygen, and thus oxygen is locally present in saturation, which increases the likelihood of ROS generation (Rutherford et al. 2012). There, photosynthetic organisms have evolved to cope with these harmful consequences of water oxidation by building the PSII in a way that minimizes the possibility of the unwanted harmful side reactions (summarized by Rutherford et al. 2012). However, photodamage can still occur, especially under high light intensities impairing the D1 protein within PSII (Krieger-Liszkay 2004; Melis 1999; Pospíšil 2016). Photosynthetic organisms came up with a complex repair mechanism that replaces the protein D1, eliminating the need to replace the entire PSII reaction center (Järvi et al. 2015; Theis and Schroda 2016). Simplified, this repair process involves the movement of PSII from its usual location in the appressed granal regions of the thylakoid membrane to the stromal lamellae, where it gets partially disassembled and reassembled with a newly synthesized D1 protein (Fig. 1A). This repaired PSII is then transferred back into the granal regions to perform its function in photosynthesis. Because the PSII is not operating normally during this repair process, the light energy absorbed by the pigments within the PSII need to be quenched to avoid ROS formation. Although the repair cycle of PSII has been a research topic for many decades now, repair intermediates are inherently difficult to study because of their relatively short lifetime and low abundancy in the crowded environment of the thylakoid membrane (Komenda et al. 2012; Nickelsen and Rengstl 2013). Thus, there are still gaps to be filled to fully understand the entire repair cycle of PSII and how ROS formation is controlled during this process.

In a recent article published in *Plant Physiology*, Fantuzzi et al. (2023) purified an assembly intermediate from the stromal lamella region of the thylakoid membrane using a fractional solubilization method (Fey et al. 2008; Haniewicz et al. 2015). Fully assembled PSII in the granal regions are usually homodimeric; however, there is a small fraction of PSII that is functional and monomeric (PSIIm). The isolated assembly intermediate from the stromal lamella in this study is similar to PSIIm, except there are 2 additional subunits present, Psb27 and PsbS (PSIIm-S/27), revealed by protein gel electrophoreses and confirmed by mass spectroscopy. Further analysis of PSIIm-S/27 showed that its pigmentation is likely identical to PSIIm. However, the fluorescence yield was substantially different, almost double the intensity compared with PSIIm. This indicates that the PSIIm-S/27 is likely not functional because the light energy gets converted into fluorescence rather than used for photosynthesis. To investigate if this is indeed the case, the authors measured oxygen evolution of both PSII monomers. And indeed, the PSIIm-S/27 showed activity that is only about 5% of that of PSIIm. To pinpoint which step within the reaction center is impaired, Fantuzzi et al. employed a series of experiments involving measurements of flash-induced chlorophyll fluorescence and Q_A_^•−^ (a plastoquinone cofactor within PSII) oxidation kinetics. This revealed that the reduction of Q_A_ to Q_A_^•−^ functions normally, but the electron transfer from Q_A_^•−^ to the mobile quinone electron acceptor at the Q_B_ site is impaired. This could potentially be caused by the binding by either or both of the 2 additional PSII proteins, Psb27 and PsbS, sterically blocking the Q_B_ binding site. To test this possibility, the authors used an inhibitor of PSII that binds to the Q_B_ site, 3-(3,4-dichlorophenyl)-1,1-dimethylurea (DCMU). If Pbsb27 and/or PsbS would occupy the Q_B_ site, addition of DCMU would not show any additional inhibitory effect on electron transfer from Q_A_^•−^ to Q_B_. However, addition of DCMU inhibited the electron transfer even further, indicating that occupation of the Q_B_ site by the 2 additional proteins is unlikely the case.

Between the Q_A_ and the Q_B_ site within the PSII, a non-heme iron is located. It has been shown that binding of bicarbonate to this non-heme iron modulates electron transfer from Q_A_^•−^ to Q_B_, stimulating forward electron transfer when present and inhibiting it when absent (reviewed by Shevela et al. 2012). It has also been shown previously that when bicarbonate is not bound, it protects the PSII from photodamage, decreasing the changes of ROS generation by “switching off” PSII (Brinkert et al. 2016). Thus, this bicarbonate could be absent in the isolated PSIIm-S/27, which would explain its loss of activity. By simply adding bicarbonate to the sample, Fantuzzi et al. were able to activate PSIIm-S/27 and showed activity comparable with PSIIm.

The authors of the article hypothesize that the isolated assembly reaction center intermediate PSIIm-S/27 occurs in the very late state of the PSII repair cycle (Fig. 1B), which is inactive, in a switched off mode that protects the reaction center from self-destruction while in transit to the granal regions. Through a yet unknown mechanism, binding of Psb27 and PsbS lowers the affinity of this assembly intermediate to bicarbonate to keep it switched off while in transit. How exactly the activation mechanism functions in vivo remains to be discovered; however, the work of Fantuzzi et al. brings us one step closer to fully understand how PSII, the engine of life on earth, is repaired and made fully functional again after having received unavoidable, self-inflicted photodamage.
